# PRECISE - pregabalin in addition to usual care for sciatica: study protocol for a randomised controlled trial

**DOI:** 10.1186/1745-6215-14-213

**Published:** 2013-07-11

**Authors:** Stephanie Mathieson, Christopher G Maher, Andrew J McLachlan, Jane Latimer, Bart W Koes, Mark J Hancock, Ian Harris, Richard O Day, Justin Pik, Stephen Jan, Laurent Billot, Chung-Wei Christine Lin

**Affiliations:** 1 The George Institute for Global Health and Sydney Medical School, University of Sydney, PO Box M201, Missenden Rd, Camperdown, Sydney, NSW 2050, Australia; 2 Faculty of Pharmacy and Centre for Education and Research on Ageing, University of Sydney, Sydney, Australia; 3 Department of General Practice, Erasmus University Medical Center, Rotterdam, The Netherlands; 4 Faculty of Human Sciences, Macquarie University, Sydney, Australia; 5 The South Western Sydney Clinical School, Faculty of Medicine, University of New South Wales, Sydney, Australia; 6 St Vincent’s Clinical School, Faculty of Medicine, University of New South Wales, Sydney, Australia; 7 ACT NeuroSpine Clinic, Australian Capital Territory, Deakin, Australia

**Keywords:** Sciatica, Pregabalin, Neuropathic pain, Randomised control trial

## Abstract

**Background:**

Sciatica is a type of neuropathic pain that is characterised by pain radiating into the leg. It is often accompanied by low back pain and neurological deficits in the lower limb. While this condition may cause significant suffering for the individual, the lack of evidence supporting effective treatments for sciatica makes clinical management difficult. Our objectives are to determine the efficacy of pregabalin on reducing leg pain intensity and its cost-effectiveness in patients with sciatica.

**Methods/Design:**

PRECISE is a prospectively registered, double-blind, randomised placebo-controlled trial of pregabalin compared to placebo, in addition to usual care. Inclusion criteria include moderate to severe leg pain below the knee with evidence of nerve root/spinal nerve involvement. Participants will be randomised to receive either pregabalin with usual care (n = 102) or placebo with usual care (n = 102) for 8 weeks. The medicine dosage will be titrated up to the participant’s optimal dose, to a maximum 600 mg per day. Follow up consultations will monitor individual progress, tolerability and adverse events. Usual care, if deemed appropriate by the study doctor, may include a referral for physical or manual therapy and/or prescription of analgesic medication. Participants, doctors and researchers collecting participant data will be blinded to treatment allocation. Participants will be assessed at baseline and at weeks 2, 4, 8, 12, 26 and 52. The primary outcome will determine the efficacy of pregabalin in reducing leg pain intensity. Secondary outcomes will include back pain intensity, disability and quality of life. Data analysis will be blinded and by intention-to-treat. A parallel economic evaluation will be conducted from health sector and societal perspectives.

**Discussion:**

This study will establish the efficacy of pregabalin in reducing leg pain intensity in patients with sciatica and provide important information regarding the effect of pregabalin treatment on disability and quality of life. The impact of this research may allow the future development of a cost-effective conservative treatment strategy for patients with sciatica.

**Trial registration:**

ClinicalTrial.gov, ACTRN 12613000530729

## Background

Neuropathic pain is a major clinical and epidemiological problem. It is a result of a lesion or disease affecting the somatosensory system [1]. The true prevalence of neuropathic pain is difficult to estimate as it encompasses a variety of disorders [2-4]. Neuropathic pain can result from a lesion in the central nervous system, such as cerebrovascular events, multiple sclerosis, spinal cord injuries, or a lesion in the peripheral nervous system, such as painful diabetic neuropathy, or post-herpetic neuralgia [5,6].

Sciatica is considered a type of neuropathic pain and can also be referred to as lumbosacral radiculopathy [7]. It is a severe form of low back pain that is characterised by radiating leg pain below the knee. Clinically, the intense leg pain may be accompanied by neurological changes of muscle weakness and wasting, sensory change and diminished reflexes in a nerve root distribution [8,9]. Sciatica is most commonly caused by lumbar disc herniation, while non-discogenic causes include bony or vascular compression, infection or malignancy [10]. Nearly a third of patients with sciatica will have persisting symptoms, continuing for up to 2 years [8,11]. Treatment strategies aim at reducing pain and preventing chronic disability, as patients with sciatica have a reduced quality of life, a longer absence from work and increased use of health resources compared to people with localised back pain [12]. Treatment may include conservative care (such as physical therapies and advice), pharmacological management and interventional procedures (such as epidural injections and surgery) [8,10,11,13,14].

There are few clinical guidelines on the treatment of sciatica, reflecting the lack of quality evidence of effective strategies [15]. We conducted a systematic review of the evidence for pharmacological management of pain in patients with sciatica and found that there was no clear evidence to support the use of non-steroidal anti-inflammatory drugs, corticosteroids, antidepressants, or opioid analgesics in the immediate term when compared to the placebo [16]. When conservative and pharmacological treatments have been unsuccessful and the leg pain persists, referral for interventional procedures such as epidural and foraminal corticosteroid injection or invasive surgery may be considered. These procedures can be costly, have no guarantee of success and come with some risk. We have found that early surgery provides a benefit in the short term but is no better than conservative care at 1 and 2 years [17,18] and that epidural corticosteroid injections provide only small, short-term benefits in pain and disability [11].

One pharmacological strategy that has been successful in the management of neuropathic pain is the use of anticonvulsant drugs [5]. Pregabalin is an anticonvulsant with analgesic and anxiolytic properties. It is a structural analogue of the neurotransmitter gamma-aminobutyric acid (GABA) that mediates its actions by binding to voltage-gated calcium channels in the central nervous system. Pregabalin is reported to provide rapid initial pain relief [7] and its lack of metabolic drug interactions means that pregabalin can be safely co-administered with other drugs [19]. Pregabalin is known to be effective in treating post-herpetic neuralgia, painful diabetic neuropathy and fibromyalgia [20-23] and recently in neuropathic components of patients with back pain [24,25] and neck pain [26]. It has also been shown to be a cost-effective alternative to usual care in patients with refractory neck pain [27].

Currently there is limited, direct, high quality research to inform the use of pregabalin in the treatment of people with sciatica. A small prospective randomised trial of patients with chronic low back pain (n = 36), which included some patients with sciatica, suggested that pregabalin may produce a statistically significant reduction in back pain in the short term, but this trial contained no placebo comparison [25]. Another study demonstrated patients had a positive response to pregabalin but the study design limited the conclusions [28]. Observational studies have reported positive results with pregabalin treatment but these trial designs contain bias [29,30]. Therefore, while there are some indications that pregabalin may be a successful treatment for patients with sciatica, high quality evidence is needed.

Sciatica is a severe, disabling condition with few effective treatment options. This double-blind, randomised, placebo-controlled trial aims to determine the efficacy, safety and cost-effectiveness of pregabalin in reducing leg pain intensity in patients with sciatica.

## Methods/Design

**Figure 1:**
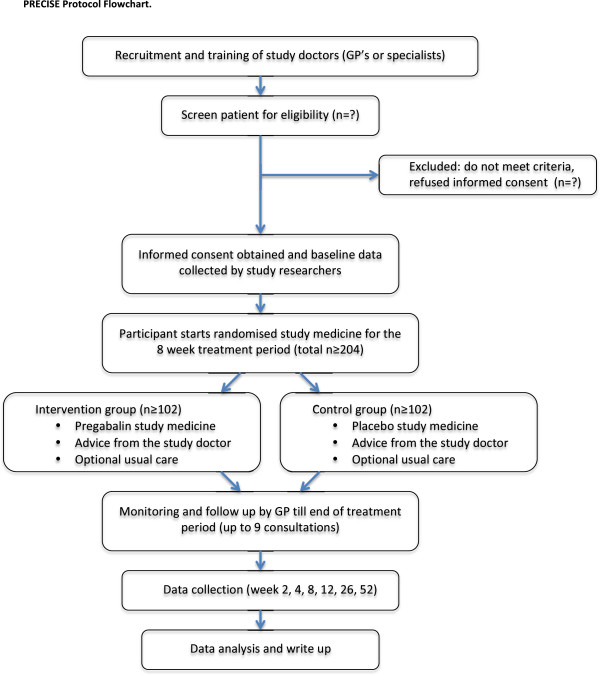
PRECISE study design.

The PRECISE study is a double-blind, randomised, placebo-controlled trial comparing pregabalin in addition to usual care, to placebo in addition to usual care for the treatment of sciatica (Figure 1). Ethics approval has been granted by the University of Sydney Human Research Ethics Committee (protocol number 15333). The study has been registered with the Australian and New Zealand Clinical Trials Registry (ACTRN12613000530729) and this study protocol follows the SPIRIT statement [31].

### Participants and recruitment

Participants will be recruited from patients in the community, who have moderate to severe sciatica, and who consult a study general practitioner (GP) or medical specialist (for example, an orthopaedic surgeon, neurosurgeon, neurologist or rheumatologist) as an outpatient [32]. GPs and specialists will be recruited from the Sydney metropolitan area (Australia) and will be required to hold current practice registration in Australia. The study doctors (that is, the GP or specialist) will obtain relevant medical history including medication history, and screen the patient against the eligibility criteria.

Eligible participants will meet all of the following criteria: radiating pain into only one leg below the knee; nerve root/spinal nerve involvement evidenced by at least one of the following clinical features: myotomal weakness, dermatomal sensory deficits, diminished reflexes, leg pain radiating in a dermatomal distribution; leg pain severe enough to cause at least moderate pain or moderate interference with normal work or daily activities over the last week (measured by adaptations of items 7 and 8 in the short form-36 (SF-36) questionnaire); pain duration of current episode of at least 1 week and up to 1 year; age 18 years or older; and sufficient understanding of the English language or interpretation assistance available to complete the study treatment and assessments.

Patients will be excluded if they meet any of the following criteria: known or suspected serious spinal pathology (such as cauda equina syndrome, or spinal fracture); pregnant or breastfeeding women, and males or females planning conception during the 8-week treatment period; scheduled or being considered for spinal surgery or interventional procedures for sciatica during the 8-week treatment period; contraindications to pregabalin (known allergy to pregabalin or significant renal impairment. Pregabalin is predominantly renally excreted, so patients with an estimated creatinine clearance of <60 ml/minute will be excluded); or already taking an anticonvulsant medication, a medication for neuropathic pain, an antidepressant or a sedative and unable to cease the medication.

If a patient is eligible, the study doctor will gain informed consent and notify the research team when a participant has been recruited by telephone, fax, email or an online system. The doctor will also administer the study treatment (see ‘Study treatment’ below), including providing the participant with a sealed medication pack (see ‘Randomisation and blinding’ below). A researcher (blinded) will collect baseline data from the participant in a telephone interview, within 72 hours of the initial treatment visit with the doctor and before the participant starts the study medication. Following baseline data collection, the researcher will inform the participant to break the seal on the medication pack and commence the study medicine. At this point the participant is considered to be randomised into the study.

To ensure consistency, the study researchers will train study doctors in the study design and conduct regular visits to confirm the protocol is being followed. We also plan to recruit patients with sciatica who present to physiotherapists and chiropractors. Registered physiotherapists and chiropractors will identify potential participants and refer them to the research team, who will then refer potential participants to a study GP for formal screening. If a patient is eligible and gives their informed consent, the study GP will then notify the research team as above and provide the study treatment.

### Randomisation and blinding

A researcher not involved in participant recruitment or data collection will generate a randomisation schedule a priori using a computer-derived random number sequence. Study medication will be prepared according to the randomisation schedule by the central study pharmacy, then sealed in an opaque medication pack and supplied to the study doctors. The randomisation schedule will be kept concealed from other researchers. Upon recruitment, the doctor (blinded) will provide a sealed medication pack to the participant (blinded), thus randomising the participant to one of two groups: pregabalin with usual care or placebo with usual care. Placebo capsules will have an identical appearance to the active pregabalin capsules. The randomisation process will ensure concealed allocation and blinding of the doctor, participant and outcome assessor.

### Study treatment

The study treatment will consist of the study medicine and advice (patient reassurance, staying active and avoiding bed rest) [33]. Each participant will receive up to nine weekly face-to-face or telephone consultations with the study doctor to begin treatment, monitor progress and adjust the dose of the study medication over the 8-week treatment period. Specialists who are unable to provide regular follow up care will refer the participant to the research team who will then arrange for a study GP to continue the 8-week study treatment.

The starting dose of the study medication, either pregabalin or placebo, is 75 mg twice daily. This will be titrated to the participant’s optimal dose, up to a maximum of 300 mg twice daily, depending on the patient’s progress and tolerability at each dose level. In the standard study dosing regimen (Table 1), we expect a 3-week titration period, then the maximum tolerated dose for each participant will be maintained for 4 weeks before the study medication is titrated down to cessation in the final (8th) treatment week. The study doctor can amend the dosage based on a participant’s adequate improvement, defined as a pain rating of 0 or 1 out of 10, for leg pain, for a minimum of 72 hours, with none or tolerable side effects. If adequate improvement is achieved before the 8-week standard treatment regimen is completed, early titration down to cessation is possible. The maximum treatment period is 8 weeks.

**Table 1:** PRECISE study medicine dosage

**Week**	**Pregabalin (or placebo) dosage**	**Total daily dose**
Week 1	1 × 75 mg capsule, twice a day, for 7 days	150 mg/day
Week 2	2 × 75 mg capsule, twice a day, for 7 days	300 mg/day
Week 3	3 × 75 mg capsule, twice a day, for 7 days	450 mg/day
Weeks 4 to 7	4 × 75 mg capsule, twice a day, for 7 days, maintained over 4 weeks *or* until adequate improvement (0 or 1 out of 10 leg pain for 72 hours)	600 mg/day
Week 8 (or after reaching adequate improvement)	Gradual titration down to cessation, for example 2 × 75 mg capsule, twice a day, for 4 days, then 1 × 75 mg capsule, twice a day, for 3 days	300 mg/day or 150 mg/day

Standard study medication regimen [5].

In addition to the study medicines, both groups may receive usual care as deemed appropriate by their study doctor during the 8-week treatment period. Usual care may include physical or manual therapies and other analgesic medications (except adjuvant analgesics). It is recommended that the study doctors follow the World Health Organisation (WHO) pain ladder [34] for analgesic medication prescription, and refrain from prescribing additional medicines for neuropathic pain or scheduling interventional procedures. Medicines for neuropathic pain include antidepressants, selective serotonin and noradrenaline re-uptake inhibitors, topical lignocaine and gabapentin and other anticonvulsant medications [7]. Also participants should not take concomitant medication that could result in an adverse interaction with pregabalin, including medicines that might increase the risk of excessive sedation (for example, benzodiazepines) [35].

### Data collection

Data collection will be conducted by the study researchers via telephone, email or online at baseline, weeks 2, 4, 8, 12, 26 and 52. Data will be collected regardless of participants’ compliance to the study treatment. Data collection by trained study researchers will minimise the study doctor workload, increase data collection quality and aid participant retention.

### Primary outcome

The primary outcome is leg pain intensity using the Numerical Pain Rating Scale, measured at baseline and weeks 2, 4, 8, 12, 26 and 52. The participants will be asked to rate their average leg pain over the last 24 hours out of 10, with zero representing ‘no leg pain’, and 10 representing the ‘worst pain imaginable’ [36].

### Secondary outcomes

The key secondary outcome is the Roland Disability Questionnaire for Sciatica [18], measured at baseline and weeks 2, 4, 8, 12, 26 and 52, to assess disability.

The Numerical Pain Rating Scale [36], rated as an average over the last 24 hours, measured at baseline and weeks 2, 4, 8, 12, 26 and 52, will assess back pain intensity.

The SF-12v2 Quality of Life questionnaire [37] will be used at baseline and weeks 2, 4, 8, 12, 26 and 52, to assess quality of life.

The Global Perceived Effect [38] will be measured at baseline and weeks 2, 4, 8, 12, 26 and 52, this asks the participant to compare their leg pain to when this episode first started. It is measured on a Likert scale, from a score of −5 (vastly worse) to 0 (unchanged), to +5 (completely recovered).

Work and health utilisation questions will be asked at weeks 4, 12, 26 and 52. These will report the use of health services and the number of hours missed from paid employment because of sciatica, for the cost-effectiveness analysis.

### Other data collected

The use of other analgesic medicines over the previous week will be collected at baseline. The PainDETECT questionnaire, which screens for the presence of neuropathic pain [39], will be completed at baseline. Confirmed pregnancy will be recorded at 2, 4, 8 and 12 weeks.

Details of adverse events will be collected at 2, 4, 8, 12, 26 and 52 weeks of treatment. The most common adverse effects of pregabalin are dizziness and somnolence [7]. Any serious adverse events, defined as an event that is life threatening, results in death, hospitalisation, or significant disability, will be reported immediately to the serious adverse events committee (authors, CM, AM and ROD). The serious adverse events committee will investigate the relationship between a serious adverse event and the study medication, and if a potential relationship is suspected, unblinding to treatment allocation is permissible. The ethics committee will also be informed of the serious adverse event.

Questions will be asked regarding blinding and satisfaction at week 8. The blinding question will ask the participant to guess to which study treatment they were randomized, that is whether they have been randomized to pregabalin or placebo, or do not know. The satisfaction question will ask the participant to rate how satisfied they felt with the study treatment overall on a 5-point scale (extremely dissatisfied, dissatisfied, neutral, satisfied or extremely satisfied).

Adherence to study medication will be documented through a self-reported daily medication diary and by counting the returned medicine, compared to the prescribed regimen as recorded by the study doctor. Participants will be asked to return unused medicines via a reply-paid post satchel at the end of the 8-week treatment period.

### Data integrity and analysis

The integrity of trial data will be monitored by regularly scrutinising data files for omissions and errors. We will perform double data entry of the primary (leg pain) and key secondary (Roland Disability Questionnaire for Sciatica) outcomes. Other outcomes will be checked using a risk-based approach, checking random samples until achieving a satisfactorily low error rate (for example, <10%). The source of any inconsistencies will be explored and resolved. Electronic data will be stored on a secure server and paper copies located in a locked cabinet. Data will only be accessible by researchers and participant confidentiality will be maintained through secure data storage, during and post-trial.

Treatment effectiveness analyses will be blinded and performed on intention-to-treat basis. A two-tailed *P*-value <0.05 will be considered statistically significant. Our primary analysis will be by analysis of covariance, to examine the effects of treatment on leg pain at week 8, using the treatment arm, baseline leg pain and symptom duration as covariates. Similar analyses will be applied to secondary endpoints. Those will be supported by longitudinal linear models of leg pain and secondary outcomes, including all available post-baseline measurements, with the baseline measurement and symptom duration as covariates. In the case of significant missing data, sensitivity analyses will be conducted where reasonable using multiple imputation [40]. A secondary analysis will assess the presence of neuropathic pain features, measured by the PainDETECT questionnaire at baseline as a modifier of treatment effects.

Economic evaluation will entail a cost-utility analysis in which the intervention will be assessed in terms of its incremental cost per quality-adjusted life-year (QALY). As we do not expect any effect on survival, our QALY estimates will be based exclusively on health state utilities. These will be obtained from measures derived from the SF-12 and transformed into health state utilities via the SF-6D algorithm [37]. For each participant, these utilities will be averaged out between observations over the entire duration of follow up of 1 year. The primary analysis will be conducted from the perspective of the health sector. The costs of the intervention will be assessed in terms of costs of medications and consultations (including follow up visits needed during titration). Healthcare services and medicines will be valued at standard rates published by the Australian Government (the Medical Benefits Scheme standard fees for non-hospital medical costs, the Pharmaceutical Benefits Schedule for costs of medicines and Australian Refined Diagnosis Related Groups cost weights for hospitalisation costs). Private non-medical healthcare services (such as physiotherapy) will be valued at standard rates published by the relevant professional body or third party payer. An additional analysis will entail a societal perspective, investigating costs associated with the use of community services (for example, community hydrotherapy classes) and work absenteeism because of sciatica. Costs of community services will be based on the self-reported costs. Costs of absenteeism from paid employment will be estimated by the number of days absent from work multiplied by the average wage rate. The incremental cost per QALY will be estimated as the ratio of the difference in average cost and QALYs between intervention arms. Sensitivity analysis will test uncertainty in key parameters such as the selection of cost weights and statistical variation in quality of life scores.

### Sample size

We have calculated the required sample size using a standard algorithm [41]. The primary outcome is leg pain at week 8. A sample size of 204 participants (102 per group) will provide 90% power to detect a difference of 1.5 out of 10 units of leg pain on the Numerical Pain Rating Scale, assuming a standard deviation of 2.5 [18], a two-tailed alpha of 0.05, and allowing for 10% of dropouts and 20% non-compliance. There is no robust evidence for the smallest worthwhile effect in pain reduction in sciatica [42]. We have based the sample-size calculation on a between-group difference of 1.5/10 in leg pain from previous trials of pregabalin for neuropathic pain [43]. The sample size also has 90% power to detect a difference between groups in score of 3 out of 23 at week 8 on our key secondary outcome, the Roland Disability Questionnaire for Sciatica. This is based on the between-group difference (3/23) and standard deviation from one of our previous sciatica trials (4/23) [18] and the same assumptions taken for the primary outcome calculation [18].

### Modification of the protocol

Any modifications to the protocol that may impact on the design and conduct of the study will require a formal protocol amendment. Such amendment will be agreed upon by the study investigators and approved by the ethics committee prior to implementation. Once approved, the changes will be communicated to the relevant parties (for example, doctors and participants).

## Discussion

This paper presents the design and rationale for a double-blind, placebo-controlled randomised trial comparing the efficacy of pregabalin and placebo in addition to usual care for patients with sciatica. If pregabalin is shown to be more effective in reducing leg pain compared to placebo, then this study will be one of the first to provide rigorous evidence of an effective conservative treatment for the management of sciatica.

The results of this research will definitively assess the efficacy and cost-effectiveness of pregabalin for the treatment of sciatica and aid in the future development of clinical management guidelines for patients with sciatica, as there are, at present, limited effective conservative treatment options.

We anticipate recruitment to start in the second half of 2013, with data collection completed by early 2016. The allocation concealment and double-blind design minimise bias, while data collection by the study researchers improves data quality and minimises the workload placed on recruiting GPs and specialists. This reduction in doctor workload will allow minimal interference with their clinical practice and facilitate recruitment. Our team has successfully conducted trials recruiting from primary care in the Sydney metropolitan area [44]. This established collaborative network and trial design will facilitate doctor and patient recruitment for a sufficient sample size, and aid compliance. Results of the study will be disseminated via publications and presentations. The completion of the PRECISE trial will potentially present a simple, effective and cost-effective treatment option for patients suffering with sciatica.

## Competing interests

The study has been awarded funding from the National Health and Medical Research Council (NHMRC), Australia. The investigators maintain full autonomy in the design, conduct and reporting of the study. We have ethics approval to reimburse study clinicians for their time spent on study specific tasks. There are no other competing interests of the trial or study sites.

## Authors’ contributions

CWCL, AJM, JL, BWK, MH, ROD and CGM conceived the study. CWCL, CGM, AJM, JL, BWK, MH, IH, ROD, JP, SJ and LB procured funding. All authors contributed to the design of the study. CWCL, CGM, AJM, JL, BWK, MH, IH, ROD form the steering committee and end point adjudication. CWCL, CM and SM form the data management team. SM and research assistants form the coordination centre, responsible for recruitment, study doctors and data collection. SM drafted the manuscript. All authors contributed to the manuscript and approved the final version for publication.
